# Theta Burst Stimulation Applied over Primary Motor and Somatosensory Cortices Produces Analgesia Unrelated to the Changes in Nociceptive Event-Related Potentials

**DOI:** 10.1371/journal.pone.0073263

**Published:** 2013-08-20

**Authors:** Diana M. E. Torta, Valéry Legrain, Maxime Algoet, Etienne Olivier, Julie Duque, André Mouraux

**Affiliations:** 1 Institute of Neuroscience (IoNS), Université catholique de Louvain, Brussels, Belgium; 2 Department of Psychology, Università di Torino, Torino, Italy; 3 Department of Experimental and Clinical Health Psychology, Ghent University, Ghent, Belgium; University of Bologna, Italy

## Abstract

Continuous theta burst stimulation (cTBS) applied over the primary motor cortex (M1) can alleviate pain although the neural basis of this effect remains largely unknown. Besides, the primary somatosensory cortex (S1) is thought to play a pivotal role in the sensori-discriminative aspects of pain perception but the analgesic effect of cTBS applied over S1 remains controversial. To investigate cTBS-induced analgesia we characterized, in two separate experiments, the effect of cTBS applied either over M1 or S1 on the event-related brain potentials (ERPs) and perception elicited by nociceptive (CO_2_ laser stimulation) and non-nociceptive (transcutaneous electrical stimulation) somatosensory stimuli. All stimuli were delivered to the ipsilateral and contralateral hand. We found that both cTBS applied over M1 and cTBS applied over S1 significantly reduced the percept elicited by nociceptive stimuli delivered to the contralateral hand as compared to similar stimulation of the ipsilateral hand. In contrast, cTBS did not modulate the perception of non-nociceptive stimuli. Surprisingly, this side-dependent analgesic effect of cTBS was not reflected in the amplitude modulation of nociceptive ERPs. Indeed, both nociceptive (N160, N240 and P360 waves) and late-latency non-nociceptive (N140 and P200 waves) ERPs elicited by stimulation of the contralateral and ipsilateral hands were similarly reduced after cTBS, suggesting an unspecific effect, possibly due to habituation or reduced alertness. In conclusion, cTBS applied over M1 and S1 reduces similarly the perception of nociceptive inputs originating from the contralateral hand, but this analgesic effect is not reflected in the magnitude of nociceptive ERPs.

## Introduction

Recent studies have suggested that cTBS, consisting of short high-frequency bursts of transcranial magnetic stimulation (TMS) delivered in a continuous fashion [1], can be applied over M1 to alleviate pain [2–4]. This analgesic effect has been attributed to a modulation of descending inhibitory systems and/or a modulation of the activity of mesial brain structures, such as the anterior cingulate and orbitofrontal cortices [3,5,6]. However, the specificity and neural basis of cTBS-induced analgesia remain largely speculative. Furthermore, although S1 is often considered as a structure playing an important role in the sensori-discriminative aspects of pain perception [5], a previous study failed to evidence any specific analgesic effect of cTBS applied over S1 [6]. Indeed, in that study, the authors reported a similar decrease in pain perception following “real” and “sham” cTBS [6]. 

Several studies have attempted to explore the neurophysiological mechanisms underlying cTBS-induced analgesia by characterizing the effect of cTBS on the magnitude of nociceptive ERPs [6–9], such as laser-evoked brain potentials (LEPs) related to the selective activation of heat-sensitive Aδ- and C-fiber skin nociceptors [10]. These studies have shown that the amplitude of nociceptive ERPs – which are thought to originate bilaterally from the insula, the secondary somatosensory cortex (S2), the anterior cingulate cortex and S1 contralateral to the stimulated side [11,12] – is reduced when cTBS is applied either over M1 [9] or over S1 [6].

Here, we investigated the respective effects of cTBS applied either over M1 or over S1 on the perception and magnitude of ERPs elicited by nociceptive and non-nociceptive somatosensory stimuli delivered to the ipsilateral or contralateral hand relative to the hemisphere onto which cTBS was applied. This allowed us to address the three following issues. First, we investigated the effect of cTBS applied over M1 or S1 on the perceptual and electrophysiological responses elicited by nociceptive and non-nociceptive somatosensory stimuli, to determine whether the modulation effect of cTBS is specific for nociception. Second, we compared the ERPs elicited by stimuli delivered to the ipsilateral vs. contralateral hand relative to the hemisphere onto which cTBS was applied in order to determine whether cTBS over M1 and S1 exerts a specific effect on the processing of sensory input originating from the contralateral hemibody. Third, by comparing the effect of cTBS on pain perception and ERP magnitude we aimed to gain a better understanding of the functional significance of nociceptive ERPs, in particular, the relationship between ERP magnitude and pain perception [13,14].

## Methods

### Participants

A total of twenty subjects participated in the present study. Nine participants (4 women, aged 29±4 years, range 22-36) took part in the first experiment (M1 experiment: cTBS applied over M1). Eleven participants (6 women, aged 30±6 years, range 22-43) took part in the second experiment (S1 experiment: cTBS applied over S1). The two experiments only differed with respect to the site on which cTBS was applied. All other procedures were identical. Participants were recruited among students and staff of the university. After giving a written informed consent, they were screened by a neurologist for contra-indications to TMS [15]. They had no history of prior neurological or psychiatric disorders and were all right-handed according to the Edinburgh Assessment [16]. All participants were naive to the aims of the study. The experimental procedures were approved by the Ethics Committee of the Université catholique de Louvain (B40320096559).

### Stimuli

Nociceptive somatosensory stimuli were 50 ms pulses of radiant heat generated by an infrared CO_2_ laser stimulator (wavelength 10.6 µm; Université catholique de Louvain). The laser stimuli were delivered to the dorsum of the left or right hand. Stimulation target was visualised using a coaxial He–Ne laser beam. Beam surface area at target site was 80 mm^2^. To avoid skin overheating and nociceptor fatigue or sensitization, the target of the laser beam was displaced to a random position on the hand dorsum after each pulse (minimum distance: 20 mm). In a preliminary session, laser stimuli of variable energy were delivered to the hand dorsum to determine, for each subject, the energy at which the stimuli elicited a clear painful pinprick sensation, detected with reaction times compatible with the conduction velocity of Aδ-fibers (<650 ms): 627 ± 59 mJ (mean ± SD, n=9) in the M1 experiment; 711 ± 84 mJ (n=11) in the S1 experiment. The same intensity of stimulation was used in the main experiment, before and after cTBS.

Non-nociceptive somatosensory stimuli consisted in non-painful 0.5 ms constant-current square-wave electrical pulses (DS7 stimulator, Digitimer Ltd, UK) delivered to the left or right median nerve at the level of the wrist using pairs of square adhesive electrodes (5x5 mm) separated by approximately 20 mm. In a preliminary session, electrical stimuli of variable intensity were delivered such as to determine the intensity required to elicit a small but consistent and visible twitch of the thumb: 4.53 ± 1.15 mA in the M1 experiment; 4.13 ± 0.97 mA in the S1 experiment. The same intensity was used before and after cTBS, and the adhesive electrodes were not displaced.

The stimulation parameters (position of the laser stimulus, timing of the electrical and laser stimuli) were computer-controlled thus preventing any experimenter-dependent bias during data acquisition.

### Procedure

**Figure 1:**
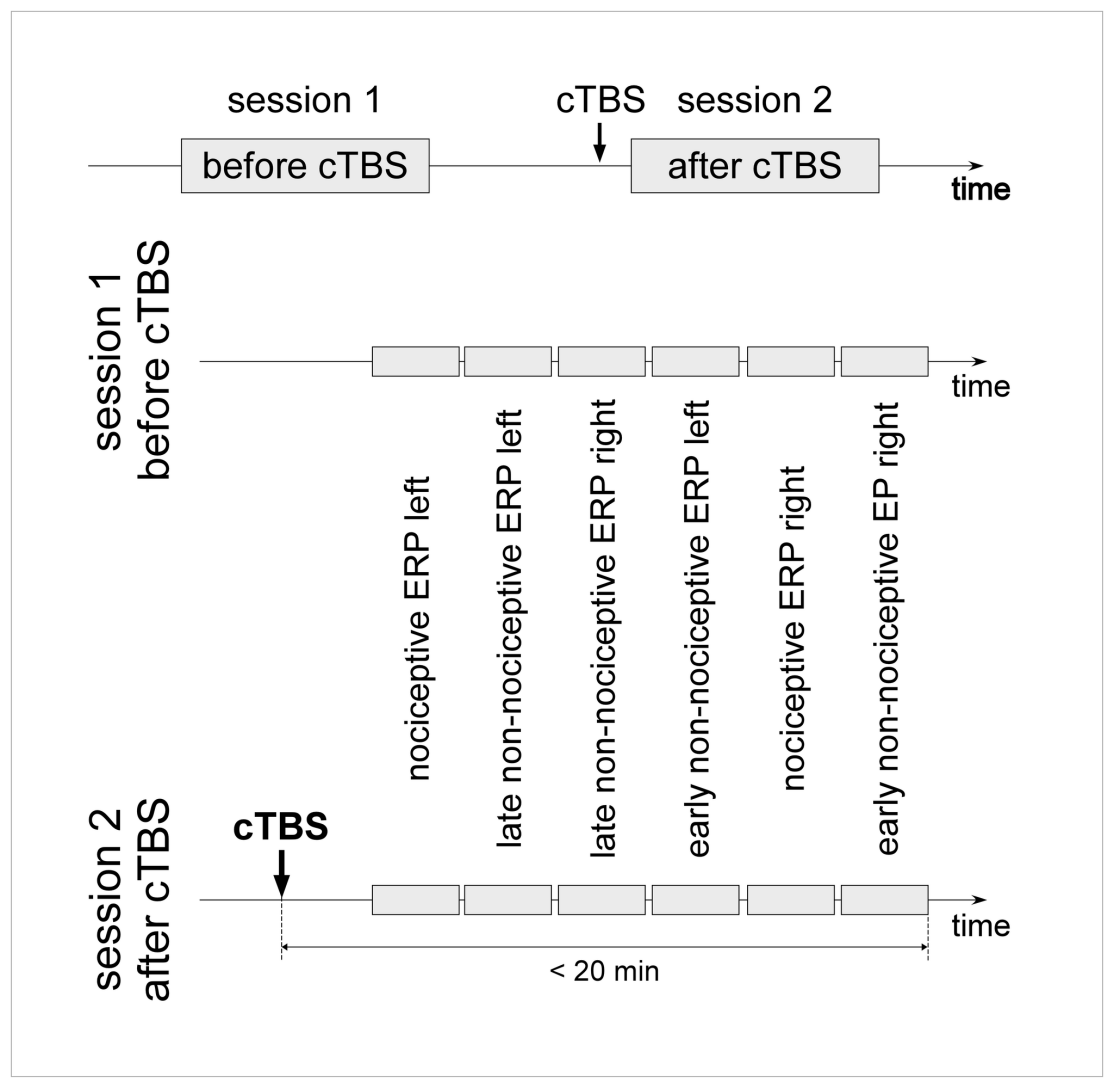
Experimental design. EEG responses to nociceptive and non-nociceptive somatosensory stimuli recorded before and after applying continuous theta burst stimulation (cTBS) over the left or right M1 or S1 cortex. The second EEG recording was always completed within 20 minutes after the end of cTBS. Nociceptive ERPs, late-latency non-nociceptive ERPs and early-latency non-nociceptive ERPs were recorded following stimulation of the left and right hand, in six separate blocks. The order of the blocks was counterbalanced across subjects, but identical in the two recording sessions.

The experiment consisted of two recording sessions separated by a cTBS session. The second recording session always began within 7 minutes after the end of cTBS and was completed within 20 minutes following cTBS application (Figure 1).

During the recording sessions, participants were comfortably seated on a reclining chair in a dimly-lit room with both arms placed on a cushion. They were instructed to keep their eyes open and to look at a fixation cross displayed on a sheet of paper positioned in front of the chair. Each experimental session began with a small practice period so as to allow subjects to become familiar with the task. Then, 6 blocks of stimuli were presented.

Two blocks of 20 nociceptive stimuli were used to elicit nociceptive ERPs (one block of stimuli delivered to the left hand, one to the right hand). In two other blocks (one for each hand), 20 non-nociceptive stimuli were delivered to assess the late components of non-nociceptive somatosensory ERPs. In these four blocks, the stimuli were delivered using a long and variable inter-stimulus interval (ISI; 5-7 s; rectangular distribution), and the participant was asked to report verbally the intensity of the elicited sensation after each stimulus using a numerical rating scale ranging from 0 (no sensation) to 10 (most intense sensation). Finally, two additional blocks (one for each hand) of 250 stimuli separated by a constant 0.25 s ISI were used to isolate the early components of non-nociceptive somatosensory ERPs. In these blocks, the participant did not rate the intensity of the stimuli. The order of the 6 blocks was counterbalanced across participants, but identical in the two recording sessions (before and after cTBS).

### Continuous theta burst stimulation (cTBS)

The magnetic pulses were generated by a Rapid Model 200 stimulator (Magstim, Whitland, UK) and delivered over M1 or S1 using a 70-mm figure-of-eight coil [17]. Both in the M1 and S1 experiments, the stimulated hemisphere (left or right) was randomized across participants to account for possible lateralization effects. In the M1 experiment, 4 subjects received cTBS over the left hemisphere and 5 subjects received cTBS over the right hemisphere. In the S1 experiment, 5 subjects received cTBS over the left hemisphere and 6 subjects received cTBS over the right hemisphere. The side of stimulation (left or right hemisphere) was not introduced as factor in the subsequent analyses. For subjects who received cTBS over the left hemisphere, sensory stimuli delivered to the left hand were labeled ‘ipsilateral’ and stimuli delivered to the right hand were labeled ‘contralateral’. Conversely, for subjects who received cTBS over the right hemisphere, stimuli applied to the right hand were labeled ‘ipsilateral’ and stimuli applied to the left hand were labeled ‘contralateral’.

This experimental design allowed us to directly compare the effects of cTBS on the responses elicited by stimulation of the ipsilateral and contralateral hand and, thereby, tease out the specific effect of cTBS on the responses elicited by stimulation of the contralateral hand from general non-lateralized, effects of cTBS and/or effects unrelated to cTBS such as habituation and changes in vigilance.

**Figure 2:**
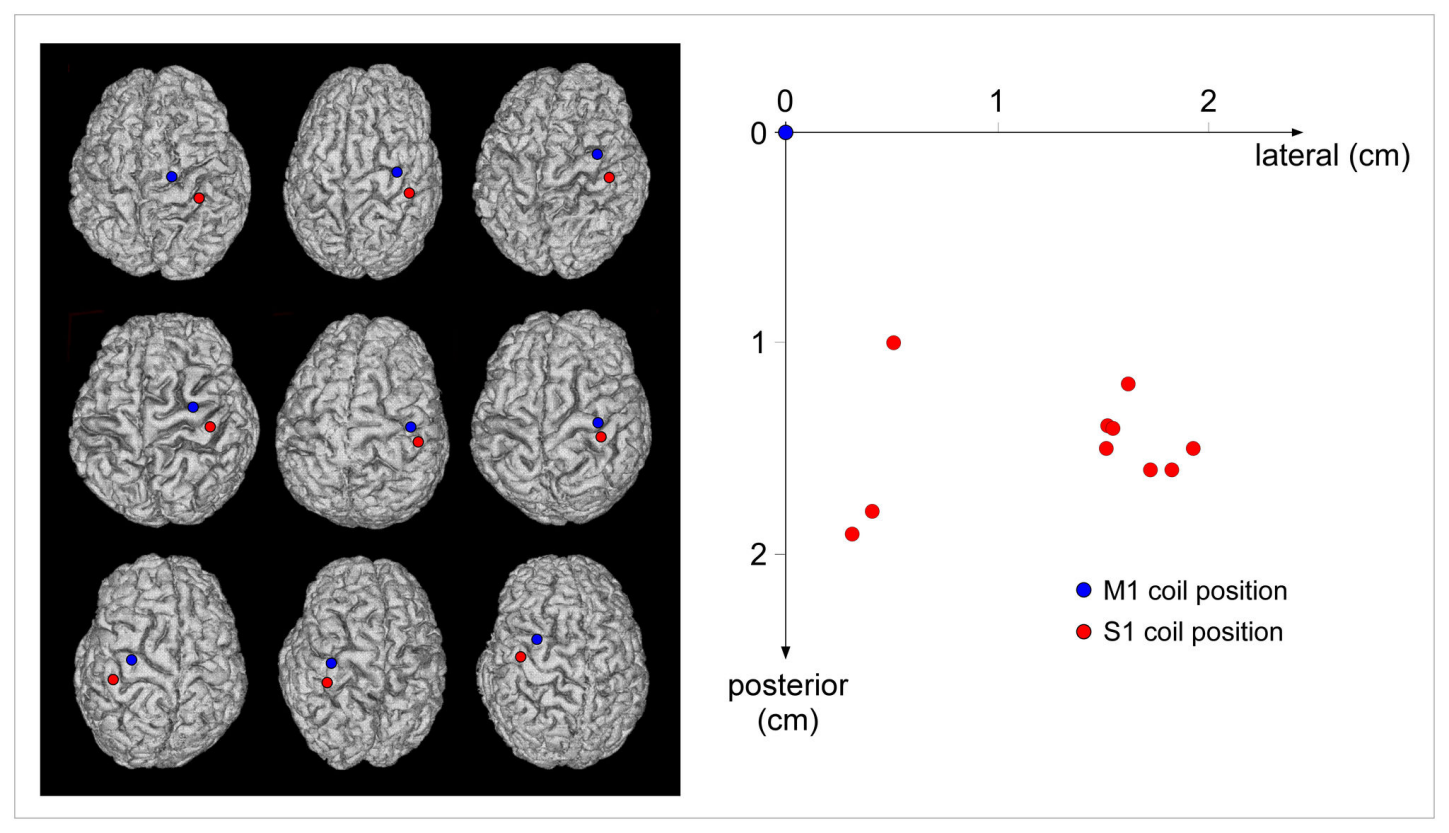
S1 coil target location in in nine representative subjects of experiment S1. Left panel. The S1 target was identified using a custom MRI-guided neuronavigation system. The position of the coil was adjusted to target the post-central gyrus at a location mirroring the M1 hotspot relative to the central sulcus, i.e. the location expected to correspond to the representation of the hand within S1. The M1 (blue) and S1 (red) targets are shown on the cortical surface reconstructed from the individual MRI data of nine representative subjects. Right panel. Using our MRI-guided approach to target S1, we found that the actual location of the coil on the scalp surface was both more posterior and more lateral relative to the M1 coil position (x-axis: medial-lateral distance relative to the M1 coil position; y-axis: anterior–posterior distance relative to the M1 coil position).

In both experiments, the handle was positioned over the targeted hemisphere, oriented towards the back of the head and laterally at a 45° angle away from the midline, approximately perpendicular to the central sulcus. After fitting the participant with a headcap (Electro-cap, Electro-Cap International, USA), the “hot spot” of the M1 hand area was identified by searching for the coil position at which single pulses slightly above threshold consistently produced the largest motor-evoked potential (MEP) in the first dorsal interosseus (FDI) muscle of the contralateral hand [18]. This location was marked on the cap to provide a reference point. The resting motor threshold (RMT) was defined at the hot spot as the minimal TMS intensity required to evoke MEPs of about 50 µV peak-to-peak in the contralateral FDI in 5 out of 10 consecutive trials. The mean RMT, expressed as percentage of maximum stimulator output, was 60 ± 14% in the M1 experiment, and 57 ± 8% in the S1 experiment. In the S1 experiment, a custom MRI-guided neuronavigation system [19] was used to co-register this position onto individual MRI data acquired before the TMS experiment. Then, the position of the coil was adjusted to target the post-central gyrus at a location mirroring the M1 hotspot with respect to the central sulcus. This location was assumed to correspond to the hand representation within S1 [20]. The location of the M1 and S1 hotspots gathered for the S1 experiment is illustrated in Figure 2.

The cTBS protocol consisted in a series of three pulses delivered at 50 Hz, repeated every 200 ms during 40 s (total number of pulses: 600) [1]. The intensity of the stimulation was set to 80% of the RMT [21].

### EEG recording

The EEG was recorded at a 4 kHz sampling rate (64-channel ASA-LAB EEG system; Advanced Neuro Technologies, The Netherlands) using 8 actively-shielded Ag–AgCl electrodes (Cz, Fz, C3, C4, T7, T8, and the left and right mastoids M1 and M2 according to the International extended 10-20 system; Waveguard; Advanced Neuro Technologies, The Netherlands). A ground electrode was positioned at FCz. Finally, two electrodes placed at the upper-left and lower-right sides of the right eye were used to monitor ocular movements and eye-blink artifacts.

### Time-domain analysis of EEG data

All EEG processing steps were carried out using BV Analyzer 1.05 (Brain Products, Germany), Letswave (http://nocions.webnode.com/letswave) and EEGLAB (http://sccn.ucsd.edu/eeglab).

#### Time-domain analysis of nociceptive and late non-nociceptive ERPs

The continuous EEG recordings were band-pass filtered using a 0.3-30 Hz Butterworth zero phase filter and segmented into 3 s epochs ranging from -0.5 to +2.5 s relative to stimulus onset. After baseline correction (reference interval -0.5 to 0 s), epochs containing signals exceeding ±100 µV were rejected. Separate average waveforms were computed for each subject, recording session (before vs. after cTBS), type of stimulus (nociceptive vs. non-nociceptive) and stimulation site (contralateral vs. ipsilateral relative to the hemisphere onto which cTBS was applied). To avoid any observer-dependent bias, the latencies and amplitudes of the peaks within each ERP waveform were obtained using an objective criterion (most negative or positive value within a defined time interval and at a given electrode).

Within the nociceptive ERP waveforms, three distinct peaks were identified: N160, N240 and P360 [22–24]. The N240 was identified as the most negative deflection occurring between 200 and 250 ms after the stimulus presentation, and the P360 as the most positive deflection following the N240. Both peaks were identified at the scalp vertex (electrode Cz) referenced to M1M2. The N160 was defined as the most negative deflection preceding the N240 at the temporal electrode contralateral to the stimulated hand (left hand: T8; right hand: T7) referenced to Fz [22,23].

Within the late non-nociceptive somatosensory ERP waveforms, two distinct peaks were identified at electrode Cz referenced to M1M2: N140 and P200 [25]. The N140 was defined as the most negative deflection occurring between 120 and 160 ms. The P200 was defined as the most positive deflection following the N140.

#### Time-domain analysis of early non-nociceptive somatosensory ERPs

The continuous EEG recordings were high-pass filtered at 0.3 Hz. An additional notch filter (50 Hz) was used to remove the contribution of electrical environmental noise. The EEG was then segmented into 200 ms epochs ranging from -50 to +150 ms relative to stimulus onset and baseline corrected using a reference interval ranging from -50 to 0 ms. Epochs containing signals exceeding 100 µV were rejected and average waveforms were computed for each subject, recording session (before vs. after cTBS) and stimulation site (contralateral vs. ipsilateral). Within the obtained waveforms, six successive peaks were identified at the central electrode contralateral to the stimulated hand (left hand: C4; right hand: C3) referenced to Fz: N20, P27, N30, P45, N60 and P100 [25].

### Time-frequency analysis of EEG data

A time-frequency (TF) representation based on the continuous Morlet wavelet transform (CWT) of EEG epochs was used to disclose non phase-locked EEG responses [26,27] triggered by the nociceptive and non-nociceptive somatosensory stimuli. The Morlet wavelet consists in a complex exponential function localized in time by a Gaussian envelope. The initial spread of the Gaussian wavelet was set to 2.5/πω0 (ω0 being the central frequency of the wavelet) [26,27]. Explored frequencies ranged from 0.3 to 30 Hz in steps of 0.3 Hz. The TF transform was applied to each single EEG epoch, and the obtained single-trial TF maps were averaged across trials. These maps, expressing the average oscillation amplitude regardless of phase, allowed identifying both phase-locked (i.e. ERPs) and non-phase-locked (i.e. event-related desynchronization and synchronization; ERD and ERS) stimulus-induced changes in EEG oscillation amplitude. For each estimated frequency and latency, amplitudes were expressed relative to baseline (pre-stimulus interval ranging from -0.5 to -0.1 s relative to stimulus onset), as follows: ER%_t,f_ = (A_t_,_f_ -R_f_)/R_f_, where A_t,f_ is the signal amplitude at a given latency t and frequency f, and R_f_ is the signal amplitude at the frequency f, averaged within the pre-stimulus reference interval [28].

To assess the significance of the relative increases and decreases of signal amplitude observed in the group-level average TF maps, for each time-frequency bin, a one-sample t-test against zero was performed using the amplitudes estimated in each participant. This yielded TF maps highlighting the regions where the EEG signal deviated significantly from baseline (p <0.05).

The TF maps were used to define three regions of interest (ROI) circumscribing the phase-locked and non-phase-locked EEG responses elicited by nociceptive (ROI-ERP: 0.1-0.5 s, 1-8 Hz; ROI-ERS: 0.1-0.5 s, 10-20 Hz; ROI-ERD: 0.5-0.9 s, 7-13 Hz) and non-nociceptive (ROI-ERP: 0.05-0.5 s, 1-8 Hz; ROI-ERS: 0.05-0.5 s, 10-20 Hz; ROI-ERD: 0.3-0.9 s, 7-15 Hz) stimuli. ROI-ERP circumscribed the phase-locked increase of signal amplitude, corresponding to the time-frequency representation of the nociceptive and late non-nociceptive ERPs identified in the time-domain average waveforms [27]. ROI-ERS encompassed a transient, early and non phase-locked enhancement of signal amplitude extending between 10–20 Hz. ROI-ERD encompassed a long-lasting reduction of signal amplitude of alpha-band EEG oscillations [14]. Within these ROIs, the average of the 20% of points showing maximum (ROI-ERP and ROI-ERS) or minimum (ROI-ERD) amplitudes was used as an estimate of response magnitude (top% approach) [26].

### Statistical analyses

The effect of cTBS applied over M1 and S1 on the intensity of perception and on the different EEG responses to nociceptive or non-nociceptive somatosensory stimulation was assessed using a mixed-model ANOVA with recording ‘session’ (before vs. after cTBS), ‘side' (somatosensory stimuli delivered to the hand ipsilateral vs. contralateral to the hemisphere onto which cTBS was applied) as within-subject factors, and ‘site’ (cTBS applied over M1 vs. S1) as between-subject factor. This model allowed us to determine whether cTBS applied over M1 and/or S1 specifically modulates the responses elicited by nociceptive and/or non-nociceptive somatosensory input originating from the contralateral hemibody (presence or absence of a specific interaction between the factors ‘session’ (before vs. after cTBS), ‘side' (stimuli delivered to the hand ipsilateral vs. contralateral to the hemisphere onto which cTBS was applied and, possibly, ‘site’ (cTBS applied onto M1 vs. S1). A Greenhouse-Geisser correction was used when appropriate. Significance threshold was set at p<0.05. When significant interactions were found, post-hoc paired t-tests were performed using Bonferroni correction for multiple comparisons (dividing the alpha value with the number of post-hoc comparisons).

## Results

### Intensity of perception

**Figure 3:**
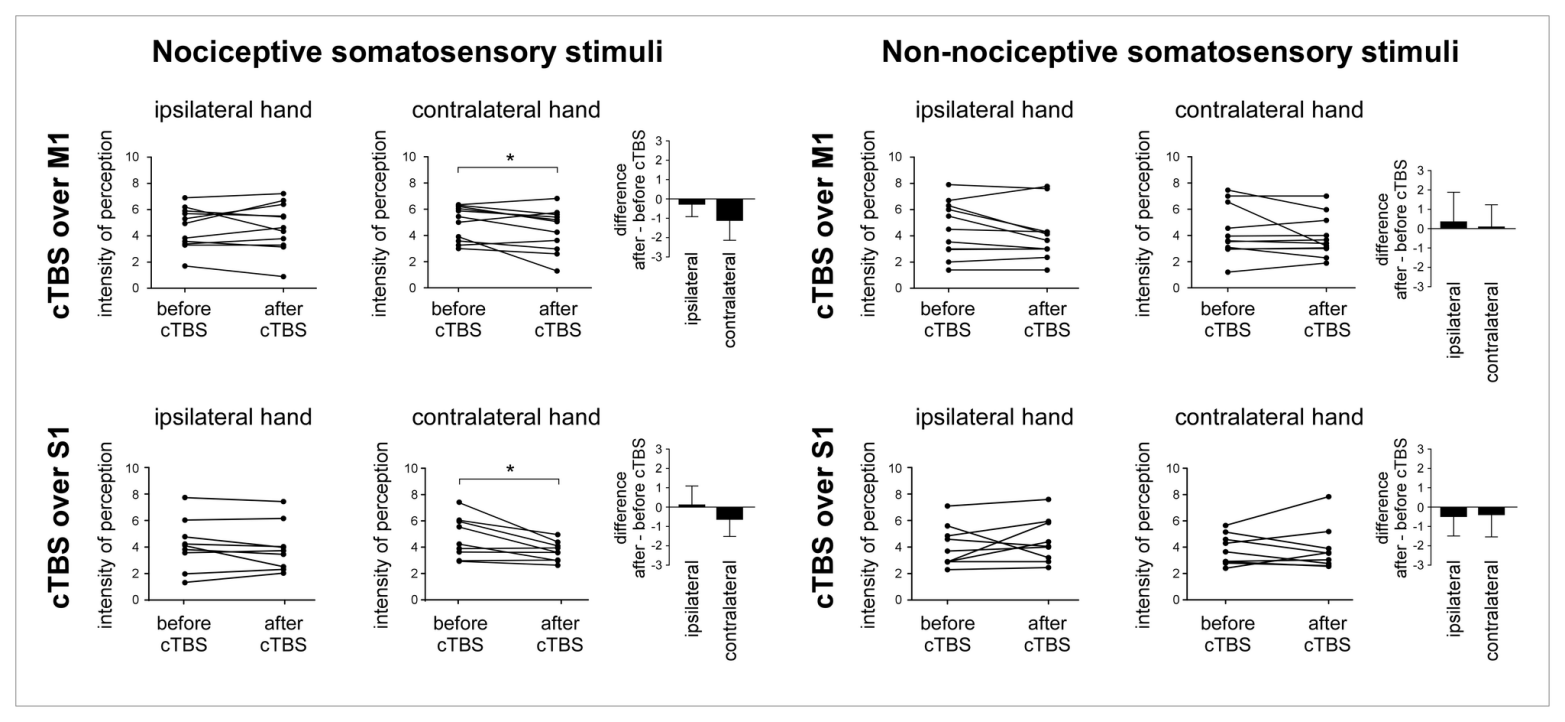
Intensity of perception. Left panel. Following cTBS applied over M1 and S1, the intensity of the percept elicited by nociceptive stimuli delivered to the hand contralateral to the stimulated hemisphere was significantly reduced. Right panel. In contrast, cTBS did not modulate the percept elicited by non-nociceptive somatosensory stimuli.

Table 1 and Figure 3 summarise the effect of cTBS on the intensity of perception.

**Table 1:** Effect of cTBS on intensity of perception.

	Nociceptive stimulus		Non-nociceptive stimulus	
	**F**	**p**	**F**	**P**
**Session**	**6.2**	.023*	0.2	.631
**session x side**	**11.2**	.004**	0.1	.761
**session x site**	1.1	.318	2.8	.112
**session x side x site**	0.2	.679	0.3	.567

cTBS applied over M1 and S1 reduced the perception elicited by nociceptive stimuli delivered to the hand contralateral to the hemisphere onto which cTBS was applied. In the M1 group, pain ratings were reduced after contralateral cTBS in 7 out of 9 volunteers (mean reduction of -17.9 ± 16.5%). In contrast, pain ratings were, on average, not affected after ipsilateral cTBS (mean change of +0.3 ± 25.0%). Similarly, in the S1 group, pain ratings were reduced after contralateral cTBS in 8 out of 11 volunteers (mean reduction of -12.5 ± 21.0%), whereas it was, on average, unaffected after ipsilateral cTBS (mean change of +0.6 ± 23.7%).

Consistently, the repeated-measures ANOVA revealed a significant interaction between the factors ‘session’ and ‘side’ (F(1,18)=11.2; p=0.004), regardless of the factor ‘site’ (Table 1 and Figure 3). Post-hoc comparisons for this significant interaction showed that, on average, cTBS applied over M1 and S1 similarly reduced the perception of nociceptive stimuli delivered to the contralateral hand (p=0.02), but left the perception of stimuli delivered to the ipsilateral hand unchanged (p=0.74).

In contrast, cTBS did not significantly modulate the perception of non-nociceptive somatosensory stimuli (Table 1 and Figure 3).

### Nociceptive ERPs

#### Analysis in the time domain

**Figure 4:**
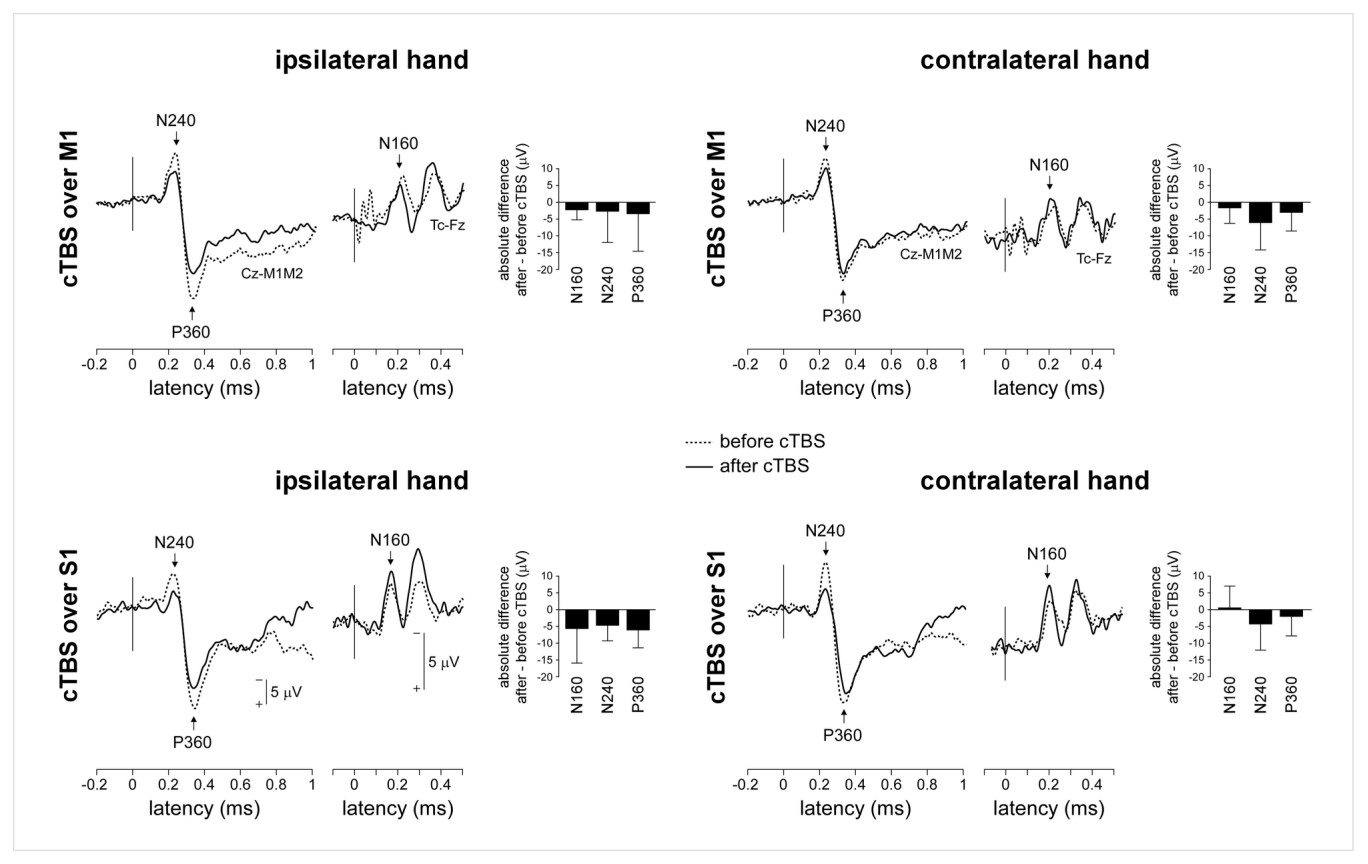
Nociceptive somatosensory ERPs (group-level average waveforms). There was no significant effect of cTBS on the magnitude of the N160 wave. In contrast, the magnitude of the N240 and P360 waves was significantly reduced following cTBS, regardless of whether the nociceptive stimuli were delivered ipsilateral vs. contralateral to the hemisphere onto which cTBS was applied.

Nociceptive laser stimuli elicited consistent N160, N240 and P360 waves in all subjects and in all experimental conditions (Figure 4, Table 2).

**Table 2:** LEP latencies and amplitudes.

N160								
	**Contralateral**				**Ipsilateral**			
	**Post cTBS**		**Pre cTBS**		**Post cTBS**		**Pre cTBS**	
	**Latency**	**Amplitude**	**Latency**	**Amplitude**	**Latency**	**Amplitude**	**Latency**	**Amplitude**
**S1**	0.207 ±0.024	-8.27±3.94	0.191 ±0.027	-8.33±3.64	0.199 ± 0.031	-12.56 ± 6.76	0.192 ± 0.018	-6.42 ± 4.68
**M1**	0.208 ±0.025	-8.50 ±5.60	0.202 ±0.025	-6.65 ±5.83	0.198± 0.026	- 10. 42± 6.87	0.194 ±0.026	-8.08 ±6.12
**N250**								
	**Contralateral**				**Ipsilateral**			
	**Post cTBS**		**Pre cTBS**		**Post cTBS**		**Pre cTBS**	
	**Latency**	**Amplitude**	**Latency**	**Amplitude**	**Latency**	**Amplitude**	**Latency**	**Amplitude**
**S1**	0.237 ±0.029	-11.06 ±8.53	0.233 ±0.022	- 15. 32 ±9.77	0.244 ±0.030	-11.64 ±10.12	0.233 ±0.028	-16.02 ±11.48
**M1**	0.238 ±0.022	-8.57 ±2.02	0.228 ±0.020	- 14. 66 ±7.84	0.234 ±0.019	-8.39 ±5.35	0.231 ±0.022	- 11. 24± 4.73
**P360**								
	**Contralateral**				**Ipsilateral**			
	**Post cTBS**		**Pre cTBS**		**Post cTBS**		**Pre cTBS**	
	**Latency**	**Amplitude**	**Latency**	**Amplitude**	**Latency**	**Amplitude**	**Latency**	**Amplitude**
**S1**	0.339 ±0.032	17.26 ±13.28	0.332 ±0.028	19.15 ±10.95	0.332 ±0.030	17.94 ±11.75	0.336 ±0.026	23.39 ±13.40
**M1**	0.355 ±0.030	18.82 ± 4.85	0.337 ±0.027	21.41 ±4.61	0.338 ±0.043	17.45 ±6.62	0.346 ±0.031	20.98 ±7.01

There was no significant effect of cTBS on the magnitude of the N160 wave (Table 3 and Figure 4). In contrast, there was a main effect of ‘session’ on the amplitude of the N240 wave (F(1,18)=12.7, p=0.002) and the P360 wave (F(1,18)=7.9, p=0.012), but no significant interaction between the factor ‘session’ and the factors ‘side’ or ‘site’ (Table 3), indicating that the N240 and P360 waves were reduced after cTBS regardless of whether the nociceptive stimuli were delivered to the ipsilateral or contralateral hand relative to the hemisphere onto which cTBS was applied, and regardless of whether cTBS was applied over M1 or S1.

**Table 3:** Effect of cTBS on the magnitude of the EEG responses to nociceptive somatosensory stimulation.

Time-domain analysis	N160		N240		P360		N240-P360	
	**F**	**p**	**F**	**p**	**F**	**P**	**F**	**p**
**Session**	2.8	.121	**12.7**	.002**	**7.9**	.012*	**30.0**	.000**
**session x side**	3.6	.081	0.4	.524	1.0	.336	0.0	.853
**session x site**	0.1	.762	0.0	.950	0.1	.801	0.0	.875
**session x side x site**	2.6	.131	0.5	.492	0.3	.572	0.7	.428
**Time-frequency analysis**	**ROI-ERP (ER%)**		**ROI-ERS (ER%)**		**ROI-ERD (ER%)**			
	**F**	**P**	**F**	**p**	**F**	**P**		
**Session**	**16.6**	.001**	**10.2**	.005*	**0.67**	**.424**		
**session x side**	0.0	.940	0.5	.513	2.85	.109		
**session x site**	0.5	.490	0.0	.948	0.277	.606		
**session x side x site**	0.8	.396	1.1	.315	0.607	.446		

There was no significant effect of cTBS on the latency of nociceptive ERPs.

#### Analysis in the time-frequency domain

**Figure 5:**
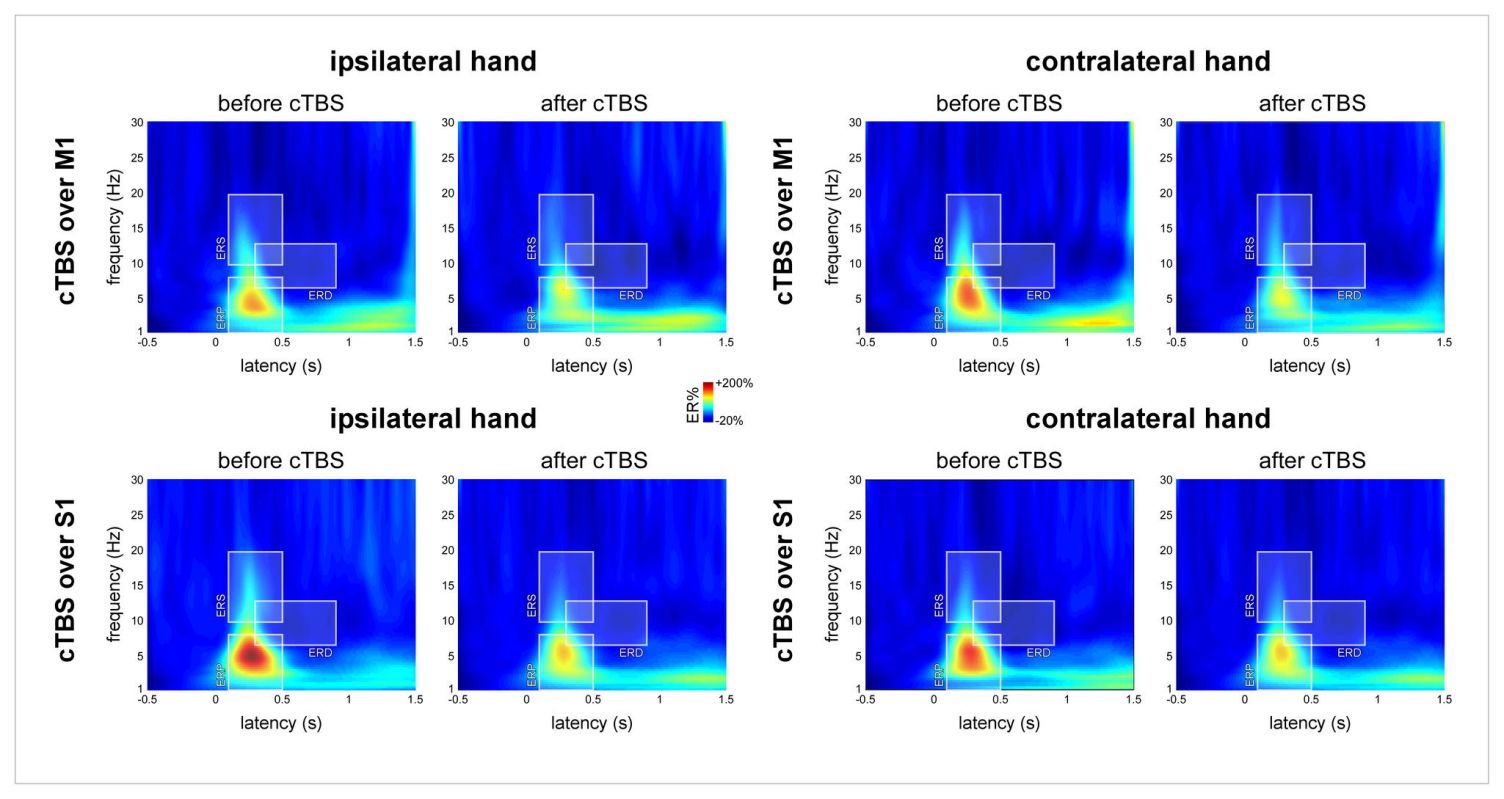
Time-frequency analysis of the EEG responses to nociceptive somatosensory stimuli. The colour maps represent the group-level average EEG signal amplitude expressed as percentage of change relative to baseline (ER%). x-axis: time (s); y-axis: frequency (Hz). Three time-frequency regions of interest were defined: ROI-ERP circumscribing the phase-locked nociceptive ERP, ROI-ERS circumscribing an early, non phase-locked enhancement of signal power between 10–20 Hz and ROI-ERD circumscribing a long-lasting desynchronization of alpha-band power. Mirroring the effect of cTBS on the magnitude of the N240 and P360 waves, the magnitude of ROI-ERP and ROI-ERS was significantly reduced following cTBS, regardless of whether the nociceptive stimuli were delivered ipsilateral vs. contralateral to the stimulated hemisphere. The magnitude of ROI-ERD was unaffected by cTBS.

In line with the results obtained in the time domain, the magnitude of ROI-ERP (circumscribing the phase-locked ERP) showed a main effect of ‘session’ (F(1,18)=16.6, p<0.001) but no significant interaction with the factors ‘side’ or ‘site’, (Table 3 and Figure 5). The magnitude of ROI-ERS (circumscribing the transient, early-latency, non phase-locked enhancement of signal amplitude between 10–20 Hz) showed a similar main effect of ‘session’ (F(1,18)=10.2, p=0.005) with no significant interaction with the factors ‘side’ or ‘site’ (Table 3). This indicates that the magnitude of ROI-ERP and ROI-ERS was reduced after cTBS regardless of whether the nociceptive stimuli were delivered ipsilateral or contralateral to cTBS, and regardless of whether cTBS was applied over M1 or S1.

The magnitude of ROI-ERD (circumscribing the long-lasting event-related desynchronization of the alpha-band, 7-13 Hz) was unaffected by cTBS (Table 3).

### Non-nociceptive ERPs

#### Analysis in the time domain

**Figure 6:**
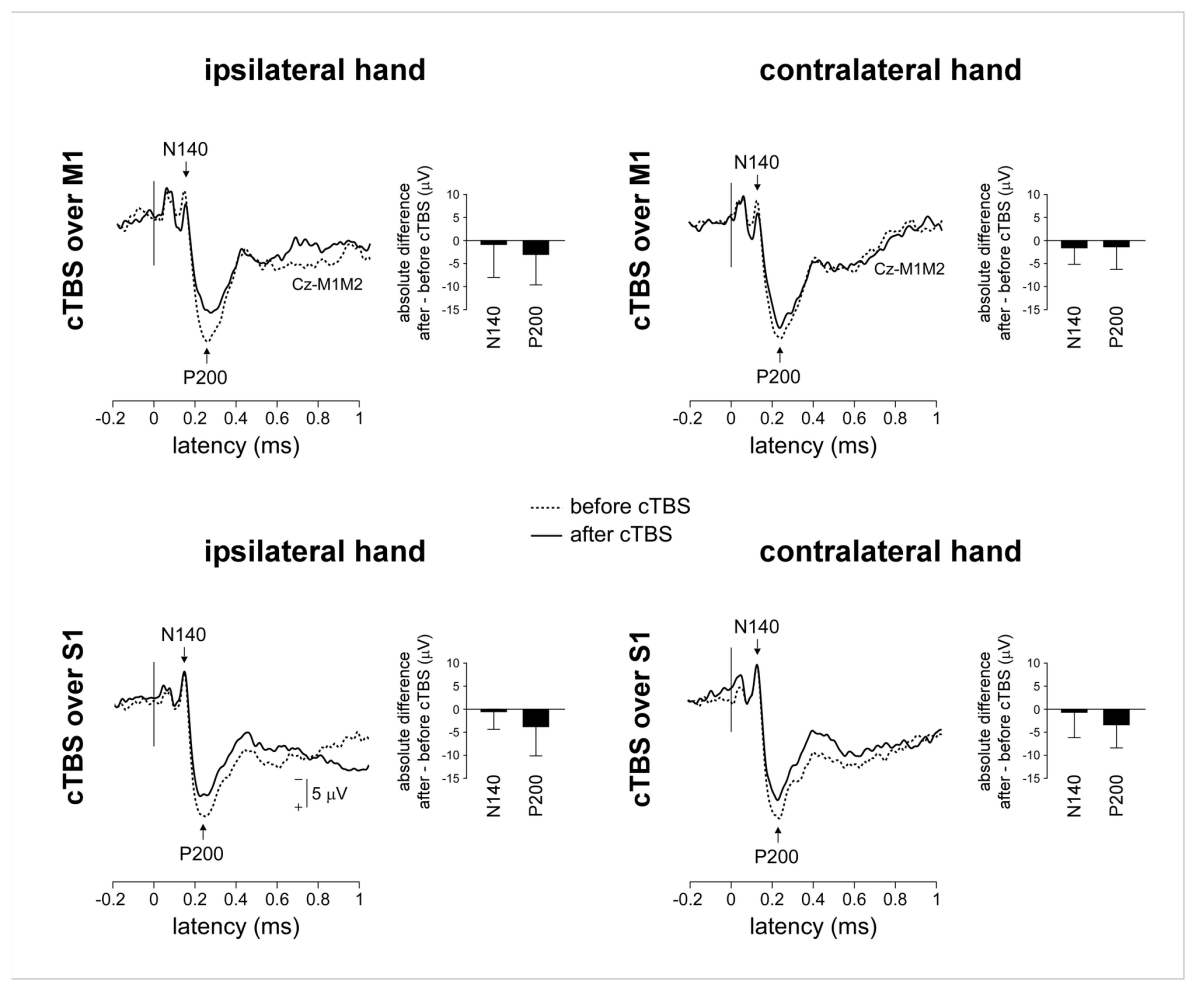
Late-latency non-nociceptive somatosensory ERPs (group-level average waveforms). There was no significant effect of cTBS on the magnitude of the N140 wave. In contrast, the magnitude of the P200 wave was significantly reduced following cTBS, regardless of whether the nociceptive stimuli were delivered ipsilateral vs. contralateral to the hemisphere onto which cTBS was applied.

Non-nociceptive stimuli elicited consistent late N140 and P200 waves in all subjects and in all experimental conditions (Figure 6).

There was no significant effect of cTBS on the magnitude of the N140 wave (Table 4 and Figure 6). In contrast, there was a main effect of ‘session’ on the amplitude of the P200 wave (F(1,18)=7.2; p=0.015), with no significant interaction between the factor ‘session’ and the factors ‘side’ and ‘site’ (Table 4), indicating that the P200 was reduced after cTBS regardless of whether the stimuli were delivered ipsilateral or contralateral to cTBS, and regardless of whether cTBS was applied over M1 or S1.

**Table 4:** Effect of cTBS on the magnitude of the late EEG responses to non-nociceptive somatosensory stimulation.

Time-domain analysis	N140		P200		N140-P200	
	**F**	**P**	**F**	**P**	**F**	**P**
**session**	0.9	.356	**7.2**	.015*	**12.7**	.002**
**session x side**	0.2	.699	0.8	.398	0.1	.718
**session x site**	0.1	.721	0.6	.459	0.2	.658
**session x side x site**	0.0	.914	0.1	.741	0.0	.863
**Time-frequency analysis**	**ROI-ERP (ER%)**		**ROI-ERS (ER%)**		**ROI-ERD (ER%)**	
	**F**	**P**	**F**	**P**	**F**	**P**
**session**	1.3	.270	0.8	.398	3.6	.074
**session x side**	0.0	.961	0.0	.497	0.3	.611
**session x site**	1.8	.196	0.4	.518	0.1	.734
**session x side x site**	0.7	.414	2.9	.108	3.2	.093

**Figure 7:**
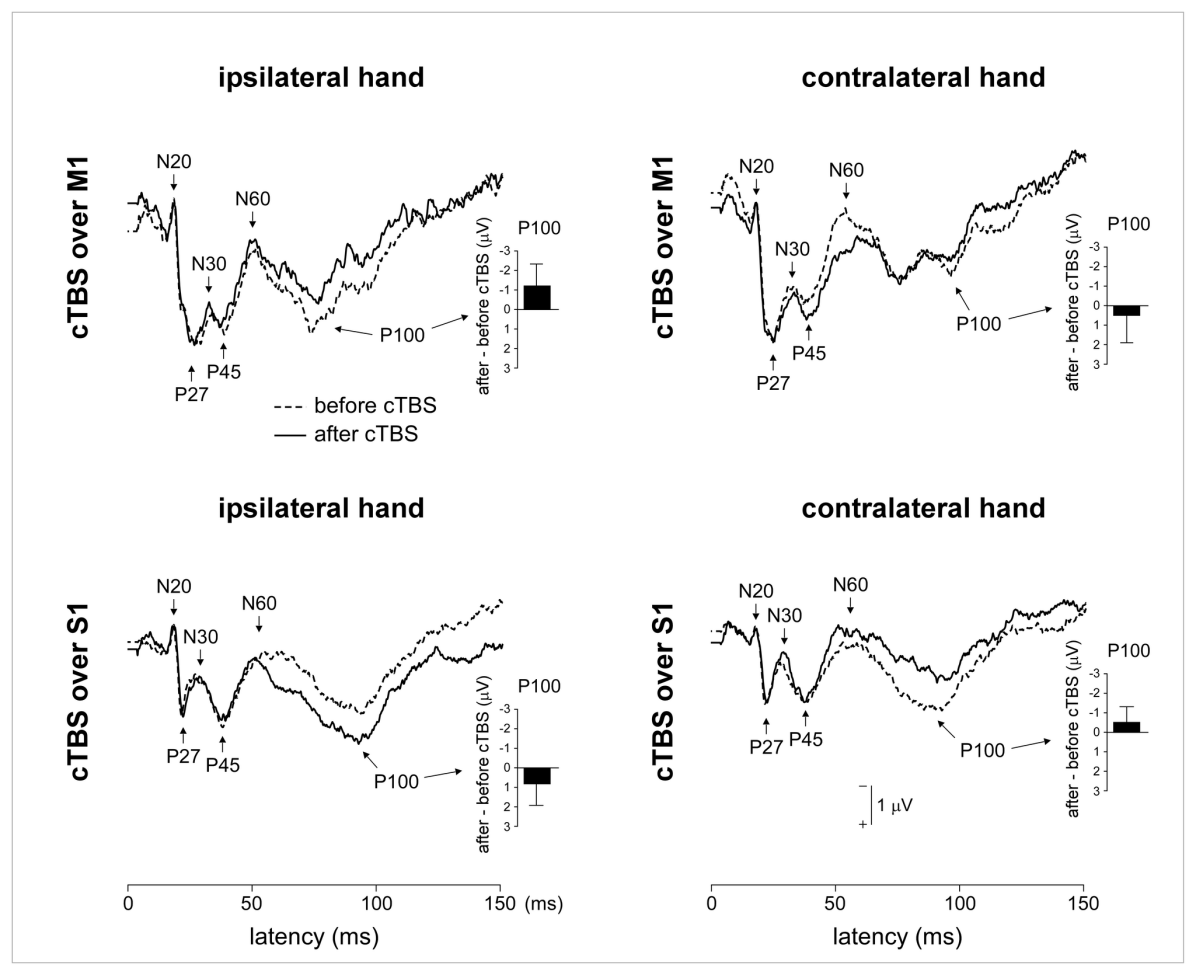
Early-latency non-nociceptive somatosensory ERPs (group-level average waveforms). There was no effect of cTBS on the magnitude of the N20, P27, N30, P45 and N60 waves. In contrast, there was a specific effect of cTBS on the magnitude of the P100 wave. The magnitude of the P100 elicited by stimuli delivered on the hand contralateral to the hemisphere onto which cTBS was applied was decreased following cTBS applied over M1 and increased following cTBS over S1. The opposite pattern was observed for the P100 elicited by stimuli delivered to the hand ipsilateral to the stimulated hemisphere. However, this observation was not confirmed by post-hoc pairwise comparisons.

Non-nociceptive stimuli also elicited consistent early-latency somatosensory ERPs (N20, P27, N30, P45, N60 and P100 waves) (Figure 7).

There was no significant effect of cTBS on the magnitude of the N20, P27, N30, P45 and N60 waves (Table 5). In contrast, there was a significant effect of cTBS on the magnitude of the P100 wave, consisting in a triple interaction between the factors ‘session’, ‘side’ and ‘site’ (F(1,18)=22.98; p<0.001). Group-level average amplitudes suggested that the P100 elicited by stimuli delivered to the contralateral hand was decreased following cTBS applied over M1 and increased following cTBS applied over S1; whereas the P100 elicited by stimuli delivered to the ipsilateral hand was increased following cTBS applied over M1 and decreased following cTBS applied over S1. However, post-hoc pairwise comparisons failed to reveal significant differences (all p >0.094).

**Table 5:** Effect of cTBS on the magnitude of the early EEG responses to non-nociceptive somatosensory stimulation.

**Time domain analysis**	**N20**		**P27**		**N30**		**P45**		**N60**		**P100**		**N20-P27**	
	**F**	**p**	**F**	**p**	**F**	**P**	**F**	**p**	**F**	**p**	**F**	**p**	**F**	**p**
**session**	0.20	.657	1.01	.328	0.87	.367	0.14	.713	0.27	.611	0.08	.789	0.06	.807
**session x side**	1.03	.325	0.25	.626	0.88	.364	0.05	.825	2.97	.106	0.23	.639	0.22	.642
**session x site**	0.24	.628	0.00	.982	1.06	.320	0.03	.861	0.32	.581	0.67	.428	0.26	.616
**session x side x site**	0.61	.446	0.67	.426	0.01	.908	0.06	.818	0.50	.490	**22.98**	.000***	3.06	.098

There was no significant effect of cTBS on the latency of non-nociceptive ERPs.

#### Analysis in the time-frequency domain

**Figure 8:**
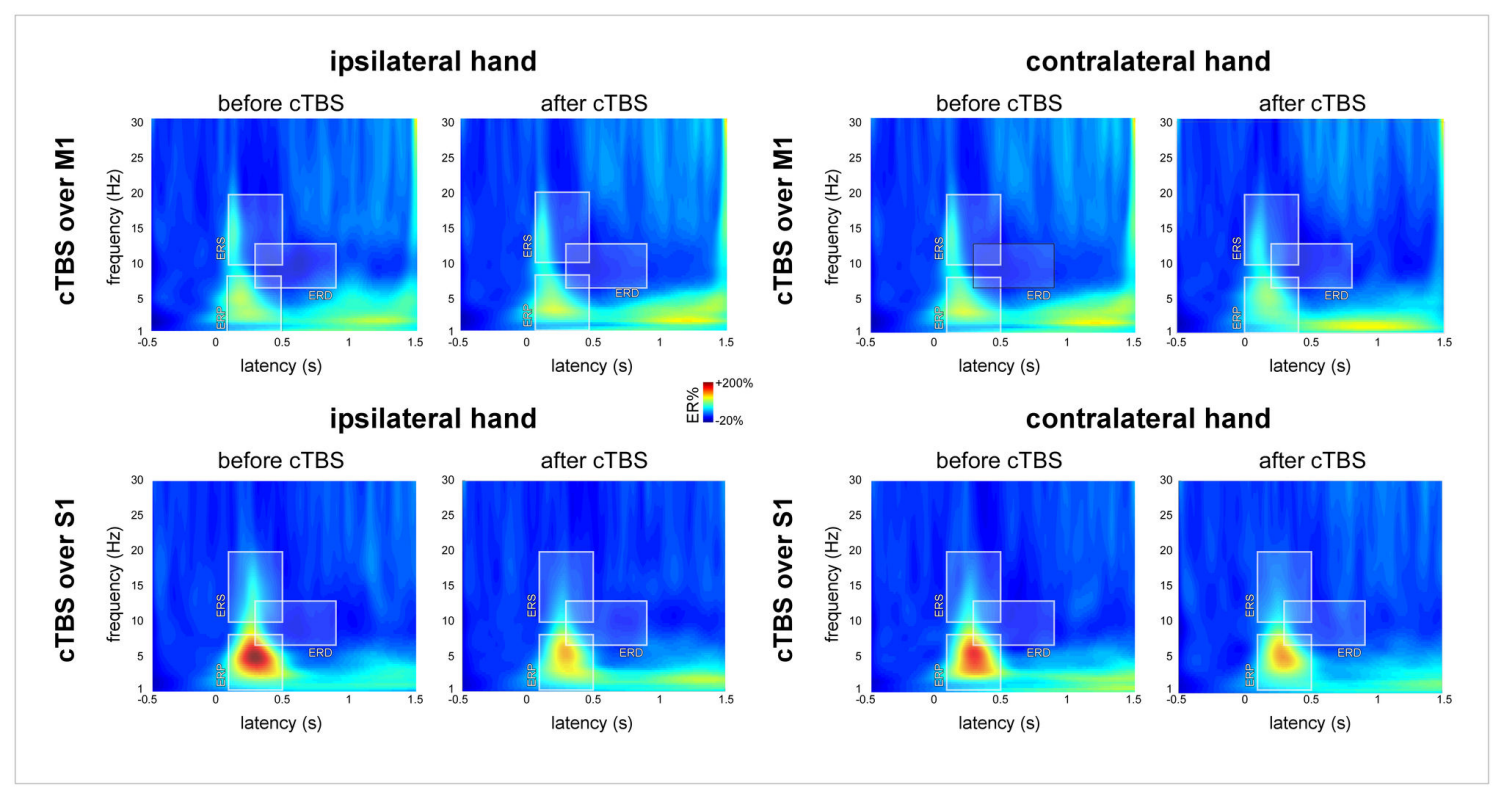
Time-frequency analysis of the EEG responses to non-nociceptive somatosensory stimuli. The colour maps represent the group-level average EEG signal amplitude expressed as percentage of change relative to baseline (ER%). x-axis: time (s); y-axis: frequency (Hz). Three time-frequency regions of interest were defined: ROI-ERP circumscribing the phase-locked ERP, ROI-ERS circumscribing an early, non phase-locked enhancement of signal power between 10–20 Hz and ROI-ERD circumscribing a long-lasting desynchronisation of alpha-band power. The magnitudes of ROI-ERP, ROI-ERS and ROI-ERD were not significantly modulated by cTBS.

The amplitude of ROI-ERP, ROI-ERS and ROI-ERD was not significantly modulated by cTBS (Table 4 and Figure 8).

## Discussion

### Effect of cTBS on the perception of nociceptive and non-nociceptive input

We found that cTBS applied over M1 as well as cTBS applied over S1 significantly reduces the perception of nociceptive stimuli delivered to the hand contralateral to the hemisphere onto which cTBS is applied, whereas it does not affect the perception of non-nociceptive somatosensory stimuli. This suggests that the perception of nociceptive input is significantly dependent on the excitability of the contralateral M1 or S1 cortex [2].

Several studies have already shown that nociceptive stimuli are perceived as less intense after the application of cTBS over the contralateral M1 [2,9]. For example, Csifcsak and colleagues [9] reported that, as compared to sham stimulation, cTBS applied over M1 results in a quantitatively greater decrease in pain ratings following stimulation of the contralateral hand as compared to stimulation of the ipsilateral hand. However, in that study, this lateralized effect on pain ratings was only significant for stimuli delivered to the right hand.

The effect of cTBS over M1 on pain perception could be explained, at least in part, by a modulation effect of TMS on remote structures [3]. For example, it has been proposed that repetitive TMS applied over M1 could indirectly reduce the responsiveness of nociceptive neurons in S2 [29] or that it could exert an inhibitory effect on the thalamus, or an excitatory effect on cingulate or orbitofrontal cortices and, consequently, on the periaqueductal grey [30–32]. According to these views, TMS over M1 would reduce the perception of pain by acting on descending pain modulatory systems and/or by acting on some cognitive and affective processes involved in pain perception [30,33,34].

Similarly to cTBS applied over M1, cTBS applied over S1 also reduced the perception of nociceptive stimuli delivered to the contralateral hand. This disagrees with the results of a previous study having shown no effect of cTBS on pain perception when applied over S1 [6]. The discrepancy between these findings can be explained by the use of different methods to position the coil over the assumed location of the hand representation in S1 [35] and/or by the stronger intensity of the TMS pulses used in the present study (80% of the rMT in the present study vs. 80% of the active motor threshold in [6]). Because a stronger TMS intensity was used in the present study, it can be proposed that the analgesic effect observed when applying TMS over S1 was, at least in part, due to a spread of the TMS-induced current to the neighbouring M1 [35,36]. However, this seems unlikely as we used a very focal 70-mm butterfly coil and as the average location of the S1 stimulation site was 1.3 cm lateral and 1.6 posterior relative to the M1 hot spot. Furthermore, using a less focal 90-mm double coil, Jacobs et al. [35] found that cTBS delivered over S1 using the same TMS configuration as in the present study enhanced rather than decreased M1 excitability.

Contrary to the effect of cTBS on pain perception, cTBS did not modulate the perception elicited by tactile stimuli. One possible explanation for this dissociation could be that the effect of cTBS on pain perception is mainly the result of a modulation of descending pathways [30,32,34] controlling the spinal transmission of ascending nociceptive inputs [37]. Indeed, such an effect on descending pain control systems would be expected to affect the perception of nociceptive inputs without affecting the perception of tactile inputs. Regarding S1, another possible explanation could be related to the fact that nociceptive and tactile inputs are not processed within the same subregions of S1. Indeed, whereas tactile inputs are primarily processed in area 3b [38], it has been proposed that nociceptive inputs are more strongly represented in area 1 [39–42], which is not only more superficial but also tangential relative to the scalp surface. Considering that the modulation effect of cTBS may be dependent on the distance and orientation of the stimulated neurons [43], one could speculate that cTBS applied over S1 does not exert the same effect on these two subregions of S1.

### Effect of cTBS on nociceptive and late non-nociceptive somatosensory ERPs

We found that both the magnitude of nociceptive ERPs (N240 and P360) and the magnitude of late-latency non-nociceptive ERPs (N140 and P200) were reduced after cTBS application either over M1 or S1. However, this reduction was similar for stimuli delivered to the ipsilateral and contralateral hand, a finding that could be related to an unspecific effect of cTBS on the elicited brain responses or to factors unrelated to cTBS such as habituation [14,44,45] or reduced vigilance [46].

Poreisz et al. [6] compared the effect of sham stimulation (coil tilted at a 90° angle relative to the scalp surface) and of different TBS protocols (including cTBS) on the magnitude of LEPs. The authors applied the stimulation over the left S1 and recorded the amplitude of LEPs elicited by stimulation of the left and right hands. They observed that the P360 wave elicited by stimulation of the ipsilateral and contralateral hand was similarly reduced following sham and TBS, indicating that this reduction in amplitude was unrelated to TBS. However, contrasting with our results, they also found that the N240 wave elicited by stimulation of the contralateral hand was significantly more reduced following TBS as compared to sham stimulation. Similarly, Csifcsak et al. [9] compared the effect of sham (TMS intensity set to 15% of the intensity used for real cTBS) and cTBS over M1 and found that LEP amplitudes were reduced after both sham and cTBS, thus indicating that at least part of the observed reduction in amplitude is unrelated to cTBS. However, for laser stimuli delivered to the contralateral hand, they also found a stronger reduction of the N240-P360 amplitude following cTBS as compared to sham. Importantly, because these studies did not include the side of stimulation (ipsilateral vs. contralateral) as a factor in their analysis, it cannot be definitively concluded that cTBS exerted a significantly different effect on the responses elicited by ipsilateral vs. contralateral stimulation.

In the present study, we also characterized the effect of cTBS on the non-phase locked EEG responses (alpha-band synchronization and desynchronization) following nociceptive and non-nociceptive somatosensory stimulation (Figures 5 and 8). As the magnitude of the ERPs identified in the time domain, these responses were similarly reduced after cTBS regardless of the stimulated side. This observation is consistent with the results of previous studies [14,44,45] suggesting that these non phase-locked EEG responses reflect cortical processes that are functionally similar to those underlying phase-locked nociceptive ERPs.

### Effect of cTBS on early-latency non-nociceptive somatosensory ERPs

We found no significant effect of cTBS on the magnitude of the N20, P27, N30, P45 and N60 waves. Previous studies on the effects of cTBS on the early-latency components of non-nociceptive somatosensory ERPs have yielded divergent findings. Some investigators [7] have reported a reduction in the magnitude of early components (P25/N30) when cTBS is applied over S1 and an increase of the magnitude of these components when cTBS is applied over M1. In contrast, other investigators failed to identify an effect of cTBS applied over S1 on these early ERP components [8]. These divergent results have been proposed to result from differences in the delay between cTBS application and the ERP recording: cTBS would exert a maximal effect on cortical excitability within the first 15 minutes after stimulation and then decay rapidly [8]. However, such an explanation seems unlikely in the present study. Indeed, the second EEG recording was always completed within 20 minutes after applying cTBS. Furthermore, during that time period, cTBS did exert a significant and specific effect on the percept elicited by the nociceptive stimuli.

Another explanation could be related to differences in the methodology used to target S1. Some studies [7,8] have arbitrarily defined the location of S1 as the position 2 cm posterior to the M1 hot spot. Other studies have defined the location of S1 by co-registering the position of the coil onto individual anatomical MRI data [6,47]. Here, we used a combination of the two approaches: MRI coregistration was used to position the coil over the post-central gyrus, at a location mirroring the functionally-defined M1 hot spot. We considered that this approach allowed us to target optimally the hand representation within S1 (Figure 2).

We did observe a specific effect of cTBS on the magnitude of the P100 component. Applied over S1, cTBS enhanced the magnitude of the P100 elicited by stimuli delivered to the contralateral hand, and decreased the magnitude of the P100 elicited by stimuli delivered to the ipsilateral hand. The opposite pattern was observed when cTBS was applied over M1. Interestingly, whereas the earlier components of somatosensory ERPs, such as the N20, P27 and P45 are thought to reflect mainly activity originating from area 3b, located within the caudal bank of the central sulcus, the P100 has been hypothesized to reflect later stages of cortical processing originating from S2 [48,49]. Hence, the differential effect of cTBS on the different components of early-latency somatosensory ERPs could be related to the fact that cTBS did not similarly modulate these different cortical regions [32].

### Dissociation between perception and ERPs

Although a large number of studies have shown that, in most circumstances, the magnitude of LEPs correlates with the intensity of pain perception (reviewed in 13,50), several studies have shown that this relationship can be easily disrupted [14,44,45]. For example, it has been reported that the repetition of three nociceptive stimuli at a short and constant inter-stimulus interval markedly reduces the magnitude of nociceptive ERPs without concurrently affecting pain intensity ratings [14,44,45]. These observations support the view that LEPs do not reflect cortical activity directly involved in pain perception but, instead, that they may reflect processes involved in the orientation of attention towards salient stimuli [50].

## Conclusion

The finding that cTBS applied over M1 and S1 reduces the perception of nociceptive inputs originating from the contralateral hand suggests a specific effect on the perception of nociceptive input originating from the contralateral hemibody. However, the finding that this analgesic effect is not reflected in the magnitude of nociceptive ERPs indicates that these brain responses reflect processes that are not directly related to the perception of pain and, hence, that nociceptive ERPs cannot be used as an “objective measure” of pain perception [13]. Notably, this does not question the usefulness of nociceptive ERPs to document the function of nociceptive afferent pathways, Indeed, even if LEPs reflect neuronal activities that are unspecifically related to pain perception, their generation still relies on the functional state of the nociceptive system, both at peripheral and central levels. 
